# DNA Methylation Dynamics and Cocaine in the Brain: Progress and Prospects

**DOI:** 10.3390/genes8050138

**Published:** 2017-05-12

**Authors:** Kathryn Vaillancourt, Carl Ernst, Deborah Mash, Gustavo Turecki

**Affiliations:** 1 Department of Psychiatry, McGill Group for Suicide Studies, Douglas Mental Health University Institute, McGill University, Verdun, QC H4H 1R3, Canada; kathryn.vaillancourt@mail.mcgill.ca (K.V.); carl.ernst@mcgill.ca (C.E.); 2 Department of Neurology, University of Miami Miller School of Medicine, University of Miami, Coral Gables, FL 33146, USA; dmash@med.miami.edu

**Keywords:** DNA methylation, epigenetics, cocaine, addiction

## Abstract

Cytosine modifications, including DNA methylation, are stable epigenetic marks that may translate environmental change into transcriptional regulation. Research has begun to investigate DNA methylation dynamics in relation to cocaine use disorders. Specifically, DNA methylation machinery, including methyltransferases and binding proteins, are dysregulated in brain reward pathways after chronic cocaine exposure. In addition, numerous methylome-wide and candidate promoter studies have identified differential methylation, at the nucleotide level, in rodent models of cocaine abuse and drug seeking behavior. This review highlights the current progress in the field of cocaine-related methylation, and offers considerations for future research.

## 1. Introduction

**Figure 1:**
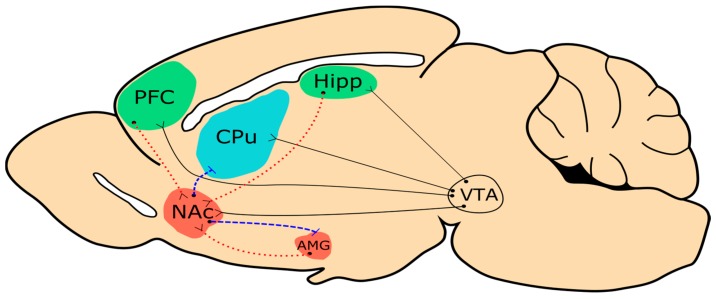
The simplified mesocorticolimbic pathway in the rodent brain. Solid black lines represent dopaminergic projection; dashed blue lines represent GABAergic projections and dotted red lines represent glutamatergic projections. Regions in green are implicated in the drug craving, blue in binge, and red in the withdrawal stages of the addiction cycle. PFC = prefrontal cortex; Hipp = hippocampus; CPu = caudate and putamen; NAc = nucleus accumbens; AMG = amygdala; VTA = ventral tegmental area.

Substance use disorders (formerly drug dependence), including cocaine use disorders, are characterized by complex behavioural symptoms, the development of physiological tolerance, and painful withdrawal symptoms [1]. Pharmacologically, cocaine is a psychostimulant that increases synaptic dopamine [2]; however, the behavioral complexity that accompanies the transition from casual drug use to cocaine dependence points to numerous, long lasting changes in cellular functioning. Researchers in this field have described an “addiction cycle” which consists of three behavioral/psychological states: binge or intoxication, withdrawal and a negative effect, and preoccupation/drug craving [3]. The development of chronic drug dependence involves the progression through the addiction cycle; alongside neuroadaptive changes to important components of the mesocorticolimbic dopamine system (Figure 1).

The striatum receives direct midbrain dopaminergic input from the ventral tegmental area (VTA), and is one of the most studied brain regions in relation to cocaine neurobiology. For example, in the nucleus accumbens (NAc), which is highly implicated in the motivational aspects of drug-seeking, repeated cocaine injections leads to increases in dendritic branching and dendritic spine formation [4,5]. These physical changes to the synaptic machinery are associated with electrophysiological adaptation throughout the addiction cycle and diverse dysregulation of RNA transcription [6,7,8]. In fact, genome-wide transcriptional changes have been found in the VTA and its projection targets, in numerous animal models of cocaine dependence and in human post-mortem tissue [9,10,11,12,13]. The specificity of these changes, with distinct networks of genes being up- or down-regulated, suggests that epigenetic mechanisms, which can be defined as covalent modifications to chromatin, may be involved.

**Figure 2:**
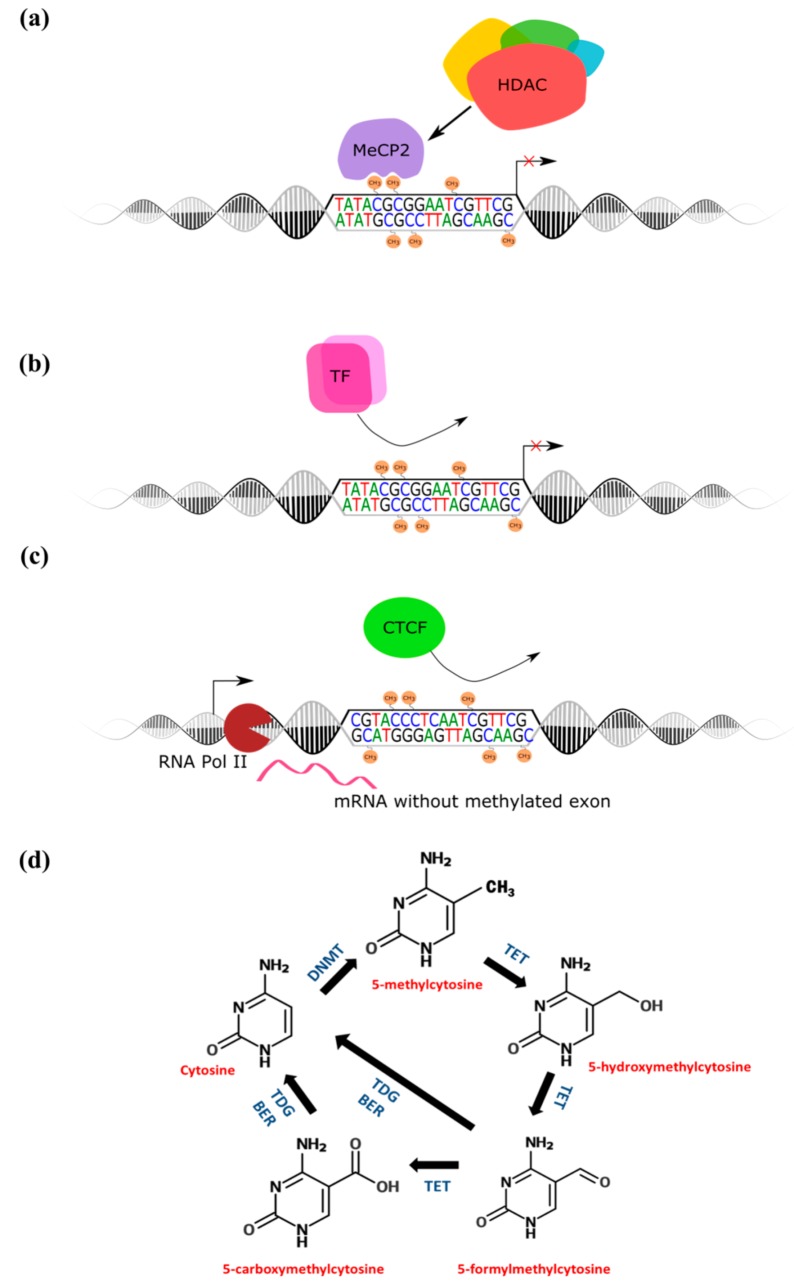
DNA methylation functions and dynamics. (**a**) methylated cytosines within gene promoters recruit methyl-binding proteins and chromatin remodeling complexes to prevent gene transcription; (**b**) methylated gene promoters prevent transcription factor binding; (**c**) exonic methylation regulated CTCF-mediated exon inclusion; (**d**) The cytosine modification cycle. MeCP2 = methylated-CpG binding protein 2; HDAC = histone deacetylase; DNMT = DNA methyltransferase; TET = ten-eleven translocation protein; TDG = thymine-DNA glycosylase; BER = base excision repair; TF = transcription factor; CTCF = CCCTC-binding factor; RNA Pol II = RNA polymerase II.

Although epigenetic mechanisms are a prominent aspect of research in developmental biology, oncology, and plant biology, the intersection between epigenetics and psychiatry is a relatively new idea. Post-translational modifications of histone tails aid in the transition between active and repressed chromatin states, and are the most well-studied epigenetic mechanisms in the context of cocaine use disorders (for review, see [14]). Cytosine modifications represent another mechanism of transcriptional regulation that have begun to gain attention in the cocaine literature. The most highly studied cytosine modification in mammals is 5′ methylated cytosine (5mC), which occurs primarily, although not exclusively, at cytosine-guanine dinucleotides (CpGs). In gene promoters, 5mC is linked to transcriptional repression, often through the recruitment of methyl-binding proteins such as MeCP2 and chromatin remodelling enzymes including histone deacetylases (HDACs) [15,16] (Figure 2a). It has also been shown that the presence of cytosine methylation within promoter sites directly represses transcription through preventing transcription factor binding, a mechanism that is particularly important in cocaine dependence [17,18] (Figure 2b). Conversely, cytosine methylation within gene bodies may promote gene expression [19,20] and recent evidence suggests that DNA methylation dynamics can negatively regulate CTCF-mediated exon translation, and alternative splicing [21,22]. In addition, 5′ hydroxymethylation (5hmC), an oxidative product of active DNA demethylation, may represent a stable epigenetic mark separate from 5mC and warrants investigation in relation to cocaine use disorders (Figure 2b) [23,24,25].

Moreover, research has directly linked neuronal activity to chromatin remodelling and epigenetic modulation [26,27,28]. In the dentate gyrus of the hippocampus, stimulation is accompanied by widespread increases in chromatin accessibility, especially around enhancer and transcription factor binding sites [26]. These changes may be a permissive, first step for active methylation changes that have been observed after stimulation [27]. Although the mechanisms through which changes in neuronal activity lead to epigenomic change are unclear, they may be mediated by intracellular calcium signalling, extracellular signal-related kinase (ERK) activity, and extracoding RNA (ecRNA) [29,30,31]. These phenomena lend further support to the notion that long term synaptic changes in the mesocorticolimbic system during cocaine dependence may be mediated by altered DNA methylation.

Indeed, the last decade saw an increase in the number of studies investigating DNA methylation and cocaine exposure, and the purpose of this review is to summarize the findings in this field. These findings can be divided into two general categories; those related to methylation machinery, the readers and writers of cytosine modifications, and those that identify differences in the presence or absence of the marks themselves.

## 2. Cocaine-Associated Alteration of DNA Methylation Machinery

### 2.1. Pharmacological Manipulations: Impact on Drug-Related Behaviors

At the broadest level, researchers have pharmacologically manipulated the DNA methylation cycle and reported diverse effects on a range of cellular and behavioral phenotypes. Overall, these studies have served to highlight the complexity of the interaction between DNA methylation and cocaine-related behaviors. For example, inhibiting DNA methyltransferase (DNMT) activity, through intracerebroventricular injections of zebularine prior to cocaine injection, delays the behavioral sensitization that is characteristic of chronic cocaine use [32]. Conversely, systemic injections of the methyl supplement S-adenosylmethionine (SAM), increases sensitization to cocaine [33]. It is important to note, however, that that the effects of pharmacological manipulations are region specific, as injections of DNMT inhibitor directly into the NAc, rather than brain-wide, enhance the sensitization phenotype after repeated cocaine injections [34].

In a similar study, Han and colleagues [35] used 5-aza-2-deoxycytidine (5-aza), to prevent de novo DNA methylation and examine the effects on the acquisition and retrieval of cocaine conditioned place preference (CPP). In this paradigm, cocaine injections are administered in a chamber with specific contextual cues and an animal’s subsequent preference for this chamber is used as a measure of the rewarding effects of cocaine. Inhibiting Dnmt activity in the hippocampus of C57BL/6 mice prior to training impaired their ability to acquire CPP, whereas the same manipulations in the prelimbic cortex prevented the retrieval of the conditioned memory after a 24 h delay [4]. Conversely, systemic injections of the methyl donor L-methionine before and throughout training, reversed the establishment of cocaine CPP altogether [36]. Interestingly, L-methionine had no effect on the establishment of CPP towards morphine or food. These studies suggest that there is a complex role of *de novo* methylation timing during the development of drug-related behavioral change, and that this role may be distinct to the cocaine context.

Another study found that daily methionine injections during ten days of intermittent cocaine exposure reduced locomotor sensitization in rats [37]. Importantly, methyl supplementation had no effect on the acute locomotor response to cocaine, which suggests that DNA methylation becomes more important as the behavioral and cellular aspects of cocaine dependence develop. To expand on this finding, Wright and colleagues [37] subjected a cohort of animals to systemic methionine injections, followed by ten days of cocaine self-administration training, and then ten days of extinction. The following day, animals were subjected to a single injection of cocaine and placed back into the self-administration chamber to measure the cocaine-induced reinstatement of drug seeking behavior. Although supplemental methionine had no effect on the establishment or extinction of cocaine-seeking behavior, animals who had received methionine injections exhibited less drug seeking behavior given cocaine during the reinstatement trial. This blunting of the reinstatement response was not seen in methionine treated animals that underwent training for sucrose pellets self-administration, which reinforces the hypothesis that DNA methylation has a more pertinent role in motivation driven behaviors to stronger, more rewarding stimuli.

Recently, Massart and colleagues [38] used an extended withdrawal paradigm, and both methylation inhibition and supplementation, to determine their role in cocaine-seeking behavior, specifically in the NAc. Intra-NAc injections of the Dnmt inhibitor RG108 in rats decreased cocaine-seeking behavior up to sixty days after the last day of cocaine self-administration. Conversely, if the animals were given intra-NAc injections of SAM, they displayed significantly more drug seeking behavior, as measured by active lever presses, than control animals up to two months post-training. In both experiments, the DNA methylation cycle was modified one month after the animals underwent CPP training, in the absence of cocaine or cocaine-related cues. This is highly indicative of the continuously active role of DNA methylation in maintaining cocaine-related memories and priming relapse-related behaviors, even in the absence of the pharmacological effects of the drug. Together, studies that have pharmacologically manipulated the methylation cycle have shown that the role of de novo methylation in cocaine-related behaviors varies, depending on the stage of behavioral acquisition, the behavior being studied, and the tissue involved.

### 2.2. DNA Methyltransferases: The Writers of DNA Methylation

In parallel to manipulating the DNA methylation cycle itself, researchers have studied alterations in the expression and function of DNMTs themselves, in numerous cocaine paradigms, and in multiple brain regions. These are enzymes that catalyze the conversion of cytosine to 5′-methylcytosine and, in mammals, can be divided into two major classes: de novo methylators of previously unmodified cytosine residues, and maintainers of methylation signatures through DNA replication. Traditionally, DNMT3a and 3b are classified as de novo methyltransferases, while DNMT1 is a maintenance methyltransferase [39,40,41,42,43], although more recent evidence has shown that all three enzymes may exhibit de novo activity [44,45].

Cocaine-related *DNMT* expression has been primarily researched in the NAc, where the expression of *DNMT* mRNA is heavily dependent on the mode of cocaine administration and the experimental time course (Table 1). For example, a single intraperitoneal injection of cocaine in mice was related to increased *Dnmt3a* transcription after acute (1.5 h) and extended (24 h) withdrawal [32]. *Dnmt3b* expression was induced 24 h after the injection, but seven consecutive days of cocaine injections had no effect on the expression of any Dnmts in this brain area. Conversely, chronic cocaine self-administration followed by 24 h of withdrawal results in the decreased mRNA expression of *Dnmt1* and *3a* in the NAc [34]. If withdrawal is extended to 28 days, this pattern is reversed, with an increased expression of *Dnmt3a*. In a similar study, Wright and colleagues [37] saw an increased expression of both Dnmt3 proteins, and a compensatory decrease in global DNA methylation levels, after cocaine self-administration followed by extinction and cue-reinstatement. Together, these studies demonstrate that the expression of canonical de novo DNA methyltransferases is dynamically regulated in the NAc in response to cocaine-seeking behavior and withdrawal.

Expression studies outside the NAc have shown that Dnmt transcription is sensitive to cocaine exposure in the extended mesocorticostriatal dopamine pathway. In the hippocampus, *Dnmt3a* is induced after a short withdrawal period from chronic cocaine injections, whereas *Dnmt3b* expression remains elevated up to 24 h after a single dose [32]. As this is the only study of methylation machinery in the hippocampus, it is difficult to speculate whether the opposing expression patterns of the two enzymes have a functional significance on the development of cocaine-related pathology. In the prefrontal cortex (PFC), one study saw increased *Dnmt3a* mRNA expression and decreased *Dnmt3b* mRNA and protein, shortly after self-administration training in mice [36]. Conversely, in rats, cocaine self-administration followed by extinction and cue reinstatement saw no change in the levels of *Dnmt* expression, nor in the overall DNA methylation in this brain area in rats [37]. These data point to a more transient role of methyltransferases in the PFC, where methylation changes are important during the acquisition phase of drug-seeking behavior, but not during the recall of drug-related memories. Importantly, all of the aforementioned studies have examined expression in whole-tissue homogenates; however, it is increasingly important to examine functional changes in diverse, independent cell types. Accumulating evidence suggests that epigenetic mechanisms, including cytosine modifications, are cell-type specific [54].

To date, two groups have reported cell-type specific changes in *Dnmt* transcription after cocaine, one in microglia and the other in striatal medium spiny neurons (MSNs). In a landmark study, Heiman and colleagues [46] used translating ribosome affinity purification (TRAP) to isolate and transcriptionally profile dopamine receptor class 1 (D1) and class 2 (D2) expressing MSNs from the striatum of cocaine-exposed BAC transgenic mice. After chronic cocaine injections, *Dnmt3a* transcription was specifically induced in D1-expressing MSNs and was accompanied by increased GABAergic activity of these cells in response to cocaine in culture. Notably, the authors found no changes in *Dnmt* expression in either MSN subgroup after a single dose of cocaine. These results emphasize the importance of cell-type specificity, and suggest that the increased methyltransferase expression seen in previous studies of striatal tissue may be occurring in specific subtypes of cells.

Glial cells are an understudied population of cells to investigate cocaine epigenetics, despite their implication in human cocaine-related transcriptional dysregulation [10,55]. In immortalized microglia from mice, even brief (3 h) exposure to cocaine *in vitro* is followed by a lasting increase in Dnmt1 and Dnmt3a protein expression [48]. Similarly, moderate doses of cocaine induce Dnmt1 protein expression in rat primary microglia, and the levels of all three classical Dnmts are increased in the microglia of mice after chronic cocaine injections. While research continues to investigate the region and cell type specificity of cocaine-related methyltransferase expression, there is also evidence to suggest that cocaine alters the expression of methylation-specific binding proteins.

### 2.3. Methyl-Binding Proteins: The Readers of DNA Methylation

Methyl-binding proteins (MBDs) bind to methylated segments of DNA, with varied specificity, and commonly recruit chromatin remodelling complexes to translate the signal of DNA methylation into transcriptional change [56]. Although there are seven members of the MBD family, methyl-CpG binding protein 2 (MeCP2) and methyl-CpG binding protein 1 (MBD1) are the only forms that have been studied in relation to cocaine. MeCP2 is highly abundant in brain tissue and mutations within its gene play a causative role in the neurodevelopmental disorder Rett Syndrome [57,58]. Classically, it binds to methylated DNA and recruits histone remodelers such as deacetylases (HDACs), although recent evidence suggests that it also has affinity for hydroxymethylated cytosines [59,60]. MBD1 is less well studied; however, evidence suggests that multiple isoforms of this transcriptionally repressive protein exist, with variable affinity to densely methylated promoters [61]. Variations in the expression and function of these proteins have begun to be examined in the context of cocaine, particularly in the dorsal striatum (DCPu), the prefrontal cortex (PFC), and the hippocampus.

The first study to report on MBDs in cocaine exposure used repeated drug injections and immunohistochemistry in rats [49]. After 10 days of cocaine injections, the number of Mecp2- and Mbd1- positive cells in the DPCu was significantly increased. This was also true for the frontal cortex and the dentate gyrus of the hippocampus. Although the authors found no change in the number of cells expressing Hdac1 or Hdac2, two binding partners of Mecp2, they saw an overall decrease in histone H3 acetylation in all three brain areas. These results provide some of the first evidence linking methyl-binding proteins to their chromatin-remodelling effects in cocaine; however, it’s important to note that these results were mirrored in a group of fluoxetine-treated animals, and therefore were not cocaine-specific.

A second study used a paradigm with differential access conditions to examine the relationship between cocaine self-administration and Mecp2 expression in rats [51]. In animals given extended access (6 h per day) to a cocaine-associated lever, the number of Mecp2 positive cells in the dorsal striatum was significantly increased. What’s more, Mecp2 positive cells tended to co-localize with the neuronal marker NeuN, and were not increased in animals given limited (1 h per day) access to drug self-administration. Lentiviral knockdown of Mecp2 within the dorsal striatum decreased cocaine-seeking behavior, as measured by the decreased number of infusions during an extended access session, and further experiments revealed the involvement of the microRNA *miR-212* in this effect. These results suggest that cocaine self-administration, particularly over an extended time period or with a higher cumulative dosage, induces the expression of Mecp2 and the activation of related neurotrophic and transcriptional pathways in the dorsal striatum [51].

In one of the only studies to differentiate between passive cocaine exposure and active drug-seeking, Pol Bodetto and colleagues [62] measured Mecp2 expression in rats exposed to either passive cocaine injections or operant self-administration training. They found no changes in *Mecp2* mRNA expression in either the CPu or the PFC in either condition; however, the number of Mecp-immunoreactive cells in the CPu and PFC was increased in both the passive and active cocaine conditions. In a parallel experiment, the authors tested the effects of a natural food reward and saw that Mecp induction only occurred in response to active reward-seeking. It appears that exposure to cocaine alone is sufficient to induce Mecp2 protein in the PFC and the CPu, and that active cocaine seeking does not significantly amplify this effect.

In light of increased Mbd expression, Mao and colleagues [52] sought to investigate altered protein activity in response to cocaine exposure. They measured levels of brain-specific phosphorylation of Mecp2 (pMecp2), which is associated with decreased repressive activity [63], in rats after a single cocaine injection. When the proteins were quantified 20 min after the drug exposure, they saw increased Mecp2 phosphorylation in the NAc; however, it took 60 min to induce significant phosphorylation in the CPu. The NAc results were consistent with an earlier study that found increased immunofluorescence of pMecp2 compared to Mecp2 two hours after a cocaine injection in mice [50]. Whether measured as mRNA expression, protein expression, or protein activation, the effects of cocaine exposure on Mbds mirror the effects on Dnmts, with measured increases in multiple brain regions in multiple exposure paradigms.

### 2.4. Methylcytosine Dioxygenases—The Modifiers of DNA Methylation

The final group of methylation-associated proteins that will be discussed here is the ten eleven translocation (TET) family of enzymes. These are a recently discovered group of enzymes involved in the oxidation of 5′-methylcytosine to 5′-hydroxymethylcytosine, and its derivatives 5′-formyl and 5′-carboxylcytosine [64,65]. These modified nucleotides are enriched in the brain and may represent an epigenetic regulator of transcription, distinct but related to classical DNA methylation [19,25,66].

To date, there has been one published study of Tet protein expression and function in relation to cocaine. Feng and colleagues [47] repeatedly injected mice with cocaine, and examined the expression of the Tet proteins in the NAc 24 hours later. They found a down-regulation of *Tet1* mRNA and protein after chronic cocaine, but not *Tet2* or *Tet3*. Using a short hairpin knockdown of *Tet1* in the NAc of behaving animals, they confirmed that decreased *Tet1* is related to increased cocaine CPP. Conversely, overexpressing *Tet1* in the NAc decreased cocaine CPP, which suggests that this protein in the NAc is an important regulator of the behavioral response to chronic cocaine exposure. Interestingly, the down-regulation of *Tet1* mRNA observed in their animal model was mirrored in the NAc of human cocaine abusers. These findings have important implications for the generalizability of animal work to human cocaine dependency and, together with the above changes in MBDs and DNMTs, beg the question of whether cocaine is associated with methylation changes at the nucleotide level.

## 3. Cocaine-Associated Dysregulation of Methylation Dynamics

### 3.1. Global and Methylome-Wide Observations

Unlike cancer phenotypes, cocaine dependence and other psychiatric disorders typically do not show broad changes in global methylation levels (Table 2). High performance liquid chromatography (HPLC) of methylation in whole brain homogenates shows no differences between cocaine treated mice and controls [67]. A more regionally defined study found that cocaine CPP was associated with a small but significant decrease in the total methylated cytosines in the PFC, but not in the NAc [36]. Interestingly, the same study showed that the effect was reversed by methionine treatment and was absent in animals who had developed CPP towards morphine or food. More recently, Feng and colleagues [47] used liquid chromatography followed by mass spectrometry (LC-ESI-MS/MS) to confirm the absence of overall methylation changes in the NAc of chronically cocaine-treated mice. In addition, their study showed no changes in the overall level of 5hmC in this region in response to cocaine, which suggests that if cocaine-related behaviors are associated with methylation changes, they are loci-dependent and not detectable at the global level.

To date, studies of the cocaine methylome have generally used enrichment techniques to collect methylated/hydroxymethylated DNA fragments [38,53]. In one study, Massart and colleagues [38] used an antibody raised against methylated cytosine, and a promoter array (MeDip-Array), to investigate methylation in the striatum and cocaine craving in rats. With their experimental design, the authors were able to interrogate promoter methylation patterns at multiple withdrawal time points after self-administration and after cue-induced reinstatement. Overall, the authors saw a hypermethylation of promoters after extended withdrawal from cocaine, and an opposing hypomethylation of promoters when cocaine-seeking was reinstated after 30 days. Although less abundant, there appear to be distinct promoters that are hypo- or hyper-methylated one day after cocaine self-administration compared to saline yoked controls. This study also included gene-wide analyses on selected genes that had been previously implicated in cocaine use and found similar dynamic patterns of methylation change throughout the withdrawal and relapse cycle. Importantly, although there was a strong overall negative correlation between promoter methylation and transcription, very few differentially methylated loci overlapped with differentially expressed transcripts. This serves to highlight the complexity of the methylation-transcription relationship and suggests that cocaine-related methylation changes may be more stably detectable than changes in transcription.

A similar study used methyl-binding protein immunoprecipitation and high throughput sequencing (MBD-seq) to profile the methylation patterns in mouse PFC after cocaine self-administration or injection, followed by acute or prolonged abstinence and a relapse test [53]. They found distinct methylation enrichment patterns in each condition, and differentially methylated regions (DMRs) that were associated with cocaine-seeking after prolonged abstinence. In general, DMRs were enriched in gene bodies and were more methylated in the cocaine group, although there were distinct loci that appeared less methylated. Importantly, only one of their validated DMRs, *Golgb1*, a Golgi-related transport protein, corresponded with changes in the overall gene expression, and both measures were decreased in cocaine animals [53]. Instead, cocaine-related DNA methylation appears to be important in regulating alternative splicing, as a number of DMRs coincided with isoform specific expression changes; an idea that fits well with findings of cocaine-related alternative splicing in other brain areas [6].

In a second PFC study, Fonteneau and colleagues [68] trained rats to self-administer cocaine, either alone or after intra-ventricular injections of DNMT inhibitors. As was seen in mice, self-administration resulted in numerous DMRs, most of which fell within gene bodies and intergenic regions. Although roughly equal numbers of hyper- and hypo-methylated sites were seen when considering relatively small differences, the ratio was skewed towards hypermethylation when considering higher effect sizes. Using a subset of DMRs that overlapped with genes, further analysis revealed that a negative correlation between methylation and gene expression can only be found for DMRs within promoter regions and that the effect is lost when considering gene body DMRs. Interestingly, the DNMT inhibited group also showed more hyper- than hypomethylation in response to cocaine self-administration, which suggests that, at least in the PFC, cocaine-related hypermethylation is more related to a decreased removal of methylation rather than an addition of methyl groups to new locations.

To address this issue, it is important to examine the dynamics of DNA demethylation after chronic cocaine. Although the paradigm and brain region differ from the Fonteneau study [68], the Feng study [47] described earlier (see Section 2.4) investigated genome-wide hydroxymethylation changes in response to cocaine. Using an antibody pulldown specific to 5hmC (hMeDip-seq), this group identified over 10,000 peaks of differential hydroxymethylation in the mouse NAc. As was the case with methylome studies [53,68], the majority of cocaine-induced changes in 5hmC occurred in gene bodies and intergenic regions. Combining this dataset with chromatin state information revealed that 5hmC changes were enriched at enhancer sites and regions that frequently switched chromatin states in the cocaine or control groups [47]. In addition, they found that 5hmC changes were enriched at regions flanking exon boundaries, and corresponded with isoform specific gene expression, which again suggests that cocaine-induced methylation dynamics are associated with alternative splicing events. Importantly, the authors probed the stability of these changes and found that increases in 5hmC could persist for up to one month after the last dose of cocaine.

Despite no changes in the global genomic 5mC or 5hmC content, the above studies have shown that cocaine induces dynamic changes in cytosine modifications at numerous sites across the genome. Researchers typically employ gene ontology or pathway analyses in order to extract functional relevance from these genome-wide data. Although it varies between studies, cocaine-related differential methylation has been associated with pathways involved in cell morphology, neuroinflammation, kinase activity, neurotransmitter-gated ion channels, and cancer [38,47]. Unfortunately, although they are helpful in organizing data sets, these analyses are limited by the availability of known gene associations, computational predictions rather than experimental data, or unknown gene functions [73]. An alternative approach to investigating cocaine-related DNA methylation has been to explore methylation changes at specific loci.

### 3.2. Differentially Methylated Loci

Locus-specific studies typically overlap with genes known to have transcriptional or functional alterations during cocaine exposure (Table 2). For example, the *Fos* family of transcription factors has been consistently implicated in mediating the relationship between cocaine-related behaviors, epigenetic changes, and cellular adaptation [18]. Accordingly, DNA methylation dysregulation has been studied at the promoters of two immediate early genes in the *Fos* family. The promoter region of *FosB* is depleted of methylation in the NAc shortly after cocaine exposure [32]. This effect is found in mice treated acutely, with a single injection of cocaine, or repeatedly, with daily injections for seven days. Similarly, the promoter region of related gene *c-Fos* contains differentially methylated CpGs in the mesocorticostriatal circuitry of mice who have undergone cocaine self-administration training, extinction, and drug-induced reinstatement [37]. In the NAc, the average methylation of this region, and the methylation of two out of twelve CpGs in particular, is decreased in cocaine-trained animals. In the PFC, there are no overall changes between groups; however, methylation at one specific CpG is increased in the cocaine group and *c-Fos* expression levels are positively correlated with drug-seeking behavior during reinstatement. Interestingly, most of the above methylation differences, whether hyper- or hypomethylated, were rescued by methionine injections throughout training. This echoes the effects of pharmacological manipulations on cocaine behaviors (see Section 2.1) and provides promising evidence for the utility of similar interventions to modulate the molecular and behavioral effects of cocaine dependence.

Cocaine-related methylation has also been measured at specific gene promoters of protein phosphatase 1 (*Pp1*) subunits. Chronic cocaine has been shown to reduce the expression of *Pp1* catalytic subunits, and the functioning of this protein and its repressor, dopamine- and cAMP-regulated neuronal phosphoprotein (DARPP-32), are necessary for behavioral and cellular responses to cocaine [62,74,75]. Methylation of the sequence flanking the 5′ end of *Pp1cβ* is enriched in the CPu of rats after chronic cocaine injections, and is associated with the increased binding of Mecp2 [62]. This is paired with decreased gene transcription, which can be reversed by inhibiting de novo methyltransferases, and decreased Pp1cβ positive cells throughout the striatum and the PFC. Notably, these effects were absent after a single dose of cocaine, which is contrary to the results of a similar study in the NAc [32]. In this study, the authors used acute and repeated cocaine injections in mice, in addition to both an enrichment- and a sequencing- based methodology, to show increased methylation of the promoter region of the *Pp1c* gene. Together, these studies demonstrate that cocaine-related changes in Pp1 expression correlate with the de novo methylation of subunit promoters in multiple addiction-relevant striatal regions.

There have been three other gene-driven methylation studies in the rodent striatum; each using methylation data to infer a mechanistic relationship between chronic cocaine exposure and other molecular changes. The first study investigated *Cdkl5*, a gene whose product is thought to interact with Dnmt1 and Mecp2, after repeated cocaine injections in rats [69]. Chronic cocaine exposure resulted in decreased *Cdkl5* gene expression and Cdkl5 expressing cells in the striatum. These changes coincided with the increased methylation of the *Cdkl5* gene immediately downstream of the transcription start site (TSS). In order to relate these findings to the known increases in Mecp2 expression after chronic cocaine [49,51], the authors explored the relationship between the two proteins and found that Mecp2 binding to the *Cdkl5* promoter is increased after chronic cocaine, and that the expression of *Mecp2* and *Cdkl5* is inversely related in vitro [69]. It is unclear whether the hypermethylation at this locus is caused by de novo methylation or the absence of Tet activity, but the epigenetic modification of *Cdkl5* may be an important negative regulator of downstream methylation changes.

The second study to examine methylation changes at specific genetic loci in the rodent striatum generated their targets based on genome-wide transcriptional changes. After chronically injecting mice with cocaine, Anier and colleagues [33] performed an expression microarray study in the NAc, and generated lists of significantly up-regulated and down-regulated genes. From there, they measured DNA methylation enrichment at three selected gene promoters, which were chosen based on their potential implications for neuroplasticity. The decreased expression of the solute carrier gene *Slc17a7* and the cholecystokinin gene *Cck* coincided with the increased enrichment of methylated DNA at their promoters. Similarly, the increased expression of the galanin neuropeptide gene *Gal* corresponds with the decreased methylation at the *Gal* promoter. Interestingly, the authors found that supplementation with repeated SAM treatment reversed the hypomethylation of *Gal*; however, it also counterintuitively reduced the hypermethylation of *Slc17a7*. In addition, the authors found decreased promoter methylation of the *Dnmt3a* and *3b* genes that is concurrent with their increased expression. These data reinforce the complexity of cocaine-induced methylation changes in the striatum and suggest that methylation may act to modify behaviour on multiple regulatory levels.

Finally, the most recent study of gene-specific methylation changes in the striatum investigated changes at the brain derived neurotrophic factor (*Bdnf*) locus [72]. Here, the authors saw an increased expression of a specific isoform of this gene, *Bdnf IV*, in the NAc of mice after CPP training. Importantly, this change appears to be specific to CPP itself, as there was no difference in its expression in non-conditioned, cocaine treated animals. After bisulfite sequencing, the authors identified hypomethylation at a single CpG site within the promoter region of *Bdnf IV* that overlapped with the binding site for the C-myb transcription factor. Despite the methylation difference occurring at a single nucleotide, the authors demonstrated increased C-myb binding at the *Bdnf IV* promoter in cocaine-conditioned animals [72]. This, along with data suggesting that *Bdnf* overexpression increases cocaine consumption [51], provides convincing evidence for a pathway between DNA methylation, neurotrophic signalling, and cocaine-related behaviors in this brain region. 

Although the majority of methylation studies have been on changes occurring in bulk striatal tissue, there has been a small focus on glial cell specific alterations. In the first study, Nielsen and colleagues [70] trained rats to self-administer cocaine for 14 days and then examined methylation changes at promoters of white matter-related genes in the corpus callosum. Although they investigated three oligodendrocyte specific genes, including the myelin-integrity-related proteins *Mbp* and *Plp1*, they only saw differential methylation at the promoter of the *Sox10* gene, which was significantly less methylated in the cocaine-trained animals after one and 30 days of forced abstinence. More recently, Guo and colleagues [48] used microglial cell lines and FACS sorted microglia from mice to investigate methylation changes at a microRNA gene. *mMiR-124* may be involved in suppressing microglial activation in response to neuroinflammation [76] and is up-regulated in microglia in response to cocaine exposure [48]. This expression change is accompanied by large increases in methylation of the *pri-miR-124a-1* and *pri-miR-124-2* gene promoters and supports the theory that DNA methylation plays a regulatory role over other, post-transcriptional regulators, in response to chronic cocaine.

### 3.3. Developmental Findings

In addition to presenting a significant health burden to adolescent and adult users, prenatal exposure to cocaine is associated with impairments in brain development and cognitive functioning that may last through the school-age years [77]. At the epigenetic level, research has begun to examine the effects of prenatal cocaine on DNA methylation within the developing rodent brain. Contrary to findings in adult animals, early work in this field identified global methylation decreases in the pyramidal layer of the hippocampus of male pups exposed to prenatal cocaine, at postnatal day three [78]. This pattern was reversed by postnatal day 30, where cocaine-exposed animals had significantly more methylated cytosine in this cell layer. Interestingly, this reversal was accompanied by site specific changes in promoter methylation, with some loci retaining their original direction of methylation change, some reversing direction, and others acquiring new methylation changes by day 30. The transition from a hypomethylated state to a hypermethylated state in this layer of cells also coincided with an increase in de novo methyltransferase expression. A similar study showed that prenatal cocaine exposure results in increased anxiety-like behaviour and impaired spatial learning in male and female offspring into adulthood [79]. Here, prenatal cocaine exposure was also associated with the hypermethylation of the paternally imprinted gene *Igf-II* and a related decrease in Igf-II protein expression in the hippocampus. Although still limited in number, these works suggest that prenatal exposure to cocaine can have long lasting repercussions on DNA methylation dynamics. It remains to be known how these changes may impact future drug-seeking behavior and related neurobiology.

## 4. Future Directions

The current body of literature on DNA methylation dynamics and cocaine is relatively young, and although it has generated interesting evidence of cocaine-associated dysregulation, many questions about the mechanisms, complexity, and generalizability of these findings remain. Notably, there is a distinct lack of research using non-rodent models, let alone human samples. The only non-rodent study in this field was performed in marmoset monkeys, and revealed the differential methylation of a single CpG within the promoter of the tachykinin receptor 3 (*TACR3*) gene, in blood [80]. While valuable, it is still unclear to what extent peripheral methylation studies can be used as markers of methylation levels in the brain [81]. Moreover, it is becoming increasingly clear that epigenetic modifications, including DNA methylation, vary between cell types within the same tissue [54,82,83]. It is important that future work in this field, whether using methylome-wide or candidate loci approaches, consider the impact of cellular heterogeneity and assess cocaine-related methylation changes in distinct cell types wherever possible.

The genome-wide data thus far have indicated that the majority of cocaine-related methylation changes occur in areas outside of gene promoters [38,53]; however, all candidate gene methylation studies have been performed in promoter regions. This discrepancy has led to gaps in our knowledge that can be addressed by methylome-wide studies using updated techniques and extensive investigations of non-promoter methylation changes. Indeed, the traditional view of the relationship between DNA methylation and transcription is becoming more nuanced, with the inclusion of hydroxymethylation and methylated gene bodies being positively correlated with gene expression and alternative splicing [84,85]. In addition, cytosine methylation outside of the CpG context has recently been found to be enriched in the adult mammalian brain and is likely to regulate transcription [86,87]; the roles of non-CpG methylation and cocaine action remain to be seen.

DNA methylation may also have roles in managing other epigenomic and higher-level chromatin architecture. For example, decreased DNA methylation and binding of associated methyl-binding proteins is associated with impairments in histone modifications and chromatin looping [88,89]. Although research has developed in each domain, using the same models and paradigms of cocaine abuse, there is little in the way of relating DNA methylation profiles to other epigenetic marks. Going forward, particularly as the mechanisms underlying the relationship between these phenomena are revealed, researchers should consider the broader implications of DNA methylation on the epigenomic landscape in order to holistically understand the molecular changes associated with cocaine use, and ultimately, human addiction.

In addition, it remains to be seen how methylation dynamics are impacted throughout the timecourse of developing drug dependency, or in specific cell types and circuits. The development of single-cell epigenomics technologies will undoubtedly be instrumental in these endeavours. To date, single cell bisulfite sequencing is able to capture methylation information for up to 50% of CpGs within a single cell and has found extensive heterogeneity, even between cells of the same type [90,91]. Furthermore, the development of CRISPR/Cas9-based epigenome editing tools, such as methyltransferases and demethylases [92,93,94,95], has generated the possibility of the direct manipulation of methylation states at specific, cocaine-related loci, and will allow for causative experiments in vitro, and eventually, in behaving animals. 

Finally, increasing attention has been given to the trans/intergenerational effects of environmental experience, including cocaine-exposure, on epigenetic phenomena. Notably, Vassoler and colleagues [96] found less cocaine-seeking behavior in male offspring of cocaine-exposed sires than in the offspring of control animals. This behavior was linked to decreased prefrontal BDNF expression in the offspring and increased *Bdnf* histone acetylation in the sperm of the sires; however, the mechanisms by which these observations are related remains unclear. Furthermore, many studies that examine “transgenerational epigenetics” in relation to behavior have not investigated behavioral or epigenetic change beyond the F1 generation [97]. We should take caution when interpreting these early studies; however, further research in this exciting field will help delineate the role of cocaine on the epigenome, beyond a single generation [98].

## 5. Conclusions

Researchers have started to investigate the relationship between DNA methylation dynamics and cocaine use disorders from multiple viewpoints. Pharmacological manipulations have shown that DNA methylation plays a dynamic role in the development of cocaine-related behaviors, and, in general, DNA methyltransferases and methyl-binding proteins are induced by numerous cocaine paradigms. At the nucleotide level, cocaine exposure is associated with diverse hyper-and hypo (hydroxy) methylation in multiple genomic contexts. The stability of methylation changes, and the growing evidence of their association with neuroadaptive changes, in addition to synaptic plasticity and memory formation (for review see [99]), makes this an exciting field of study.

To date, our knowledge of cocaine-related methylation changes comes from studies using diverse drug exposure paradigms and brain regions. Given what has been shown in the NAc, it seems likely that early cocaine exposure results in dynamic and locus-specific changes in 5mC and 5hmC alongside immediate early gene expression. As drug-seeking behaviours become more compulsive, and the extended dopaminergic circuitry is recruited, perhaps DNA methylation will gain a more homeostatic role in maintaining synaptic change. In order to develop a comprehensive understanding of genome-wide methylation changes in specific cell types during the phases of the addiction cycle, it will be imperative for researchers to embrace emerging technologies. With time, studies will undoubtedly fill the gaps in our knowledge on the impact of cocaine dependence on the methylome, the translatability of these findings to human patients, and the place of DNA methylation in the broader cocaine epigenome.

## Figures and Tables

**Table 1:** Cocaine-associated dysregulation of DNA methylation machinery.

Ref	Species *	Tissue **	Paradigm	Withdrawal	Summary Finding
*Methyltransferases and Dioxygenases*					
[46]	M	Striatum	Injection	4 h	↑ *Dnmt3a* mRNA in D1-MSNs only after chronic injection
[33]	M	NAc; Hipp	Injection	24 h	acute injection ↑ *Dnmt3a/b* mRNA in NAc and *Dnmt3b* in Hipp
					repeat injection had no effect on *Dnmt* expression
				1.5 h	acute injection ↑ *Dnmt3a* mRNA in NAc and *Dnmt3b* in Hipp
					chronic injection ↑ *Dnmt3a* mRNA in Hipp
[34]	M	NAc	Self Admin	24 h or 28 days	Biphasic expression of *Dnmt3a* mRNA (↓ 24 h but ↑ 28 days withdrawal)
			Injection	28 days	↑ DNMT3a mRNA
[36]	M	PFC	Self Admin	2 h	↑ *Dnmt3a* mRNA and ↓ *Dnmt3b* mRNA and protein
[47]	M	NAc	Injection	24 h	↓ *Tet1* mRNA and protein
	H	NAc	Post mortem	N/A	↓ *TET1* mRNA
[37]	R	NAc; PFC	Self Admin	0 h	↑ *Dnmt3a/b* mRNA expression in NAc only
[48]	M	BV-2 cells	24 h Exposure	N/A	↑ Dnmt1 and Dnmt3a protein
	R	Microglia	24 h Exposure	N/A	↑ Dnmt1 protein and all *Dnmt* mRNA
	M	Microglia	Injection	1 h	↑ all *Dnmt* mRNA
*Methyl-Binding Proteins*					
[49]	R	DCPu; FC; DG	Injection	15 h	↑ Mecp2 and Mbd1 positive cells in all areas
[50]	M	NAc	Injection	2 h	↑ phosphorylated Mecp2
[51]	R	DCPu; PFC; Hipp	Self Admin	24 h	↑ Mecp2 protein and positive neurons in DCPu with extended access
					↓ Mecp2 protein a in PFC with extended access
					↑ Mecp2 protein in Hipp with restricted or extended access
[52]	R	CPu; Nac; PFC	Injection	20 min	↑ Mecp2 phosphorylation in NAc
				60 min	↑ Mecp2 phosphorylation in CPu
[53]	R	CPu; PFC	Injection/Self Admin	5-15 h	↑ Mecp2 expressing cells with no change in mRNA

* M = mouse; R = rat; H = human; ** NAc = Nucleus Accumbens; CPu = caudate and putamen; DCPu = dorsal caudate and putamen PFC = prefrontal cortex; FC = frontal cortex; Hipp = hippocampus; DG = dentate gyrus.

**Table 2:** Cocaine-associated dysregulation of methylation dynamics.

Ref	Species *	Tissue **	Paradigm ^#^	Withdrawal	Technology ^##^	Summary Finding
*Global Methylation Level*						
[36]	M	NAc; PFC	CPP	Not reported	HPLC	Slight decrease in 5mC in PFC
[67]	M	WB	Injection	1 h	HPLC	No differences
[47]	M	NAc	Injection	24 h	LC-ESI-MS/MS	No differences in 5mC nor 5hmC
*Cocaine Methylome*						
[53]	M	PFC	Injection Self-Admin	0–2 h	MBD-seq	distinct patterns of DNA methylation after active and passive cocaine
						29 persistent DMRs after self-administration (↑ 24 and ↓ 5)
[47]	M	NAc	Injection	24 h	hMeDip-seq	5hmC alterations at enhancer regions and alternative spliced sites
[38]	R	NAc	Self Admin	1 or 30 days	MeDip-Array	Dynamic (hypo and hyper-) methylation after 1 day withdrawal
						Locus-specific enhancement or reversal of early methylation changes after 30 days
						Cue-reinstatement reversal of many withdrawal induced changes
[68]	R	PFC	Self Admin	24 h	MBD-seq	More hyper- than hypomethyated DMRs at higher differential methylation ratios
*Differentially Methylated Loci*						
[32]	M	NAc	Injection	24 h	MeDip Bis-qPCR	↑ *PP1c* promoter methylation after acute and chronic cocaine
				1.5 h	MeDip-qPCR	↓ *fosB* promoter methylation after acute and chronic cocaine
[69]	R	Striatum	Injection	15 h	Bis-seq	↑ *Cdkl5* promoter methylation
[37]	R	NAc	Self Admin	0 h	Bis-seq	↓ *c-Fos* promoter methylation
[70]	R	CC	Self Admin	30 days	Bis-seq	↓ *Sox10* promoter methylation
[33]	M	NAc	Injection	24 h	MeDip-qPCR	↑ *Scl17a7* and *Cck* promoter methylation and ↓ *Gal, DNMT3a and DMNT3b* promoter methylation
[71]	R	CPu	Injection	12 h	MeDip-qPCR	↑ *PP1Cβ* promoter methylation after chronic cocaine
[48]	M	Microglia	Injection	1 h	Bis-seq	↑ *Mir124* promoter methylation
[72]	M	NAc	CPP	2 h	Bis-seq	↓ *BDNF IV* promoter at single CpG

* M = mouse; R = rat; ** NAc = Nucleus Accumbens; CPu = caudate and putamen; PFC = prefrontal cortex; WB = whole brain; CC = corpus callosum; ^#^ CPP = conditioned place preference; ^##^ HPLC = high performance liquid chromatography; LC-ESI-MS/MS = liquid chromatography-electrospray ionization tandem mass spectrometry; MBD-seq = methyl-binding protein capture–sequencing; MeDIP = methylated DNA immunoprecipitation; hMeDIP = hydroxymethylated DNA immunoprecipitation; Bis-seq = sodium bisulfite conversion-sequencing; qPCR = quantitative polymerase chain reaction.
